# A Comparison of Conventional and Technology-Mediated Selection Interviews With Regard to Interviewees’ Performance, Perceptions, Strain, and Anxiety

**DOI:** 10.3389/fpsyg.2020.603632

**Published:** 2021-01-12

**Authors:** Klaus G. Melchers, Amadeus Petrig, Johannes M. Basch, Juergen Sauer

**Affiliations:** ^1^Institut für Psychologie und Pädagogik, Universität Ulm, Ulm, Germany; ^2^Migros-Genossenschafts-Bund, Zurich, Switzerland; ^3^Department of Psychology, University of Fribourg, Fribourg, Switzerland

**Keywords:** selection interviews, technology-mediated interviews, interview performance, personnel selection, applicant perceptions, interview anxiety

## Abstract

Organizations increasingly use technology-mediated interviews. However, only limited research is available concerning the comparability of different interview media and most of the available studies stem from a time when technology-mediated interviews were less common than in the present time. In an experiment using simulated selection interviews, we compared traditional face-to-face (FTF) interviews with telephone and videoconference interviews to determine whether ratings of interviewees’ performance, their perceptions of the interview, or their strain and anxiety are affected by the type of interview. Before participating in the actual interview, participants had a more positive view of FTF interviews compared to technology-mediated interviews. However, fairness perceptions did not differ anymore after the interview. Furthermore, there were no differences between the three interview media concerning psychological and physiological indicators of strain or interview anxiety. Nevertheless, ratings of interviewees’ performance were lower in the technology-mediated interviews than in FTF interviews. Thus, differences between different interview media can still be found nowadays even though most applicants are much more familiar with technology-mediated communication than in the past. The results show that organizations should take this into account and therefore avoid using different interview media when they interview different applicants for the same job opening.

## Introduction

Over the past decades, technological progress has considerably changed how organizations recruit and select applicants (Tippins and Adler, 2011; Ryan et al., 2015; Ployhart et al., 2017). The computer and telecommunication technology now available allows organizations to use web-based and computer-administered selection tools at all stages of the selection process. Furthermore, given the COVID-19 pandemic, many organizations had to change their selection processes to web-based or technology-mediated procedures to be able to evaluate candidates even during times of physical distancing. To do so, rather diverse tools have been introduced such as multimedia simulation tests that are administered via the internet (Oostrom et al., 2010), internet-based testing that can be completed using a computer (Tippins, 2009) or even on a smartphone (e.g., Arthur et al., 2018), and many other procedures (cf. Tippins and Adler, 2011; Ryan et al., 2015; Woods et al., 2019).

The increasing use of technology-based selection is also accompanied by significant changes in how selection interviews are administered with organizations increasingly making use of technology-mediated interviews. Furthermore, there is not only an increase in the number of interviews that are administered via the internet, but even before the COVID-19 pandemic the number of interviews that are conducted via telephone has risen compared to earlier levels from the last millennium (Amoneit et al., 2020). This increased use of technology-mediated selection interviews in addition to, or instead of, the traditional face-to-face (FTF) interviews, raises important questions concerning the comparability of the different ways in which interviews can be conducted. However, although these questions have been previously examined, most of the relevant studies were conducted when telephone-based interviews were less common than nowadays and when easy-to-use videoconference systems like Skype, Google Hangouts, or Zoom were not commonplace. Hence, it is likely that participants in previous studies may not have been as familiar and comfortable with these systems as they may be now. Consequently, it is unclear whether these earlier results still generalize to current generations of applicants.

The purpose of the present study is to respond to repeated calls (e.g., Huffcutt and Culbertson, 2011; Levashina et al., 2014; Ryan and Ployhart, 2014; Blacksmith et al., 2016) for more research on technology-mediated interviews and to compare FTF interviews with telephone and videoconference interviews on a broad range of variables. Ratings of interviewees’ performance (i.e., of their answers to specific interview questions and/or to the interview as a whole) are central in this regard. However, interviews do not only have a diagnostic function (i.e., to identify the best applicant for a given job), but also serve an important recruiting function because organizations aim to present themselves in an attractive way regarding the applicants (Dipboye et al., 2012; Wilhelmy et al., 2016). Therefore, it is also important to examine interviewee perceptions of the different kinds of interviews.

## Background

### Selection Interviews

Meta-analytic evidence has shown that selection interviews can be highly valid selection tools that may even reach similar levels of criterion-related validity as tests of general mental ability (e.g., Huffcutt and Arthur, 1994; Huffcutt et al., 2014). However, such high levels of validity can only be obtained when structured interviews are used. In a seminal article, Campion et al. (1997) discussed several factors that affect the degree of interview structure and reviewed the existing related literature. These factors can be categorized as being related to *consistency* (e.g., asking the same questions to all applicants, all interviews are conducted by the same interviewer), *question sophistication* (e.g., asking questions that minimize probing and prompting of the interviewee), and *evaluation standardization* (e.g., using descriptively-anchored scales to rate the interviewees’ responses to each question or interviewers receiving training about how to rate applicant performance, cf. Chapman and Zweig, 2005).

Traditionally, interviews have been conducted face-to-face. However, as a consequence of the advancement of telecommunication technology – and recently also of the COVID-19 pandemic – a growing number of organizations do not only rely on traditional FTF interviews but also make use of technology-mediated interviews. These interviews can be administered via telephone or videoconference systems (Chapman et al., 2003) or they might even be conducted without an actual interviewer when organizations use asynchronous video interviewing technology. In these asynchronous video interviews, interviewees are shown the interview questions on the computer screen, record their answers with their webcam, and submit them via an online platform, so that the videotaped answers can be evaluated later (e.g., Brenner et al., 2016; Langer et al., 2017).

If one uses different administration media for different applicants who apply for the same job, this reduces the consistency with which the interview is administered and thus influences the degree of standardization. However, this factor was not yet considered by Campion et al. (1997). Furthermore, as pointed out by Huffcutt et al. (2011) in their model on interviewee performance, technology-mediated interviews might increase interviewees’ apprehensiveness when they are not familiar with a given interview medium (also cf. Lukacik et al., 2020). Furthermore, Huffcutt et al. (2011) raised the question whether using technology-mediated interviews impairs the identification of social cues that are sent from the interviewer. Therefore, it is an important question to determine whether the administration medium influences ratings of interviewees’ performance in the interview, interviewees’ perceptions of the interview, or their reactions to the interview procedure.

### Relevant Theories in the Context of Technology-Mediated Interviews

With regard to technology-mediated selection interviews, several general theoretical approaches are relevant that have been developed to describe preferences and the suitability of different media for communication with others. The first of these theories is media richness theory (e.g., Daft and Lengel, 1986), which is concerned with communication media (e.g., email, telephone, or videoconference) and the extent to which these media allow or limit the transmission of information. The theory assumes that the use of different channels of information transmission (verbal, non-verbal, and para-verbal) reduces the ambiguity of a message and thus also the uncertainty of the communication partners. There have been concerns that the conveyance of (subtle) social cues is impaired during technology-mediated communication (e.g., Straus and McGrath, 1994; Chapman and Webster, 2001; Huffcutt et al., 2011). However, the extent to which such social cues can be perceived depends on the kind of technology used. While videoconferencing represents the upper end of communication bandwidth in technology-mediated communication (since it conveys auditory and visual information but also non- and para-verbal cues), telephone conversations may be positioned in the middle of the continuum while email communication would be at the lower end of it. Based on the media richness theory, one would expect that high bandwidth communication would be more advantageous in selection interviews (even though it should be acknowledged that low bandwidth communication might be beneficial in some other situations, e.g., Sauer et al., 2000). Accordingly, FTF interviews would be more beneficial than videoconference interviews because of the more comprehensive use of different channels of information transmission and videoconference interviews would be more beneficial than telephone interviews, which allow for the most limited information transmission.

Similar to media richness theory, social presence theory (Short et al., 1976) can also be used to explain the effects associated with computer-mediated communication. It assumes that communication media differ in the level to which communication partners experience the presence of each other, including the perception of the conversation partner’s gestures, gaze, and facial expressions. Furthermore, higher social presence is associated with more positive communication outcomes such as mutual attraction, trust, and enjoyment (e.g., Lee et al., 2006). In line with this, a study by Croes et al. (2016), for example, found that face-to-face communication led to more social presence, which in turn influenced the level of interpersonal attraction.

Social presence theory makes similar predictions as media richness theory but explains the phenomena in a different way. Thus, the importance of the degree of psychological awareness of another person is stressed rather than the level of communication bandwidth. With regard to selection interviews, FTF interviews should be the most beneficial medium in terms of perceived social presence. Although the interviewee is in a different location than the interviewer both during videoconference interviews and telephone interviews, both conversation partners see each other in videoconference interviews and are probably able to perceive more social presence, whereas social presence in telephone interviews is limited to the voice.

A third theoretical approach that helps to differentiate between interview media is Potosky’s (2008) framework of media attributes. According to Potosky, media communication can vary in four general attributes: social bandwidth, interactivity, transparency, and surveillance. Social bandwidth means that communication is easier in general when more communication paths are used. Therefore, this attribute is relatively similar to what is assumed in media richness theory. Interactivity defines the extent of interaction that is possible during an interview. Transparency refers to the fact that one is aware of technology-mediation during the interview. And surveillance describes the feeling that an interview could be surveilled or recorded by a third party. The first three attributes are beneficial in the context of technology-mediated interviews and the fourth attribute is negative.

Taken together, all three theoretical approaches agree that there are several advantages of FTF interviews compared to both kinds of technology-mediated interviews. This is either because of the most complete transmission of subtle cues (according to media richness theory), because of the actual presence of the conversation partners (according to social presence theory) or because of the highest level of transparency and the lowest risk of surveillance (according to Potosky’s media attributes). Furthermore, according to all the different approaches, videoconference interviews should be more advantageous than telephone interviews because the lack of visual information in the latter might make it more difficult to correctly interpret ambiguous social cues and might also impair the social nature of the interview. Furthermore, the lack of non-verbal behavior should result in interaction impairments (because non-verbal signals cannot be used as additional sources of conversation information) and impairments of transparency (see also Morelli et al., 2017).

In addition to theories that were developed to describe the suitability of different media for communication with others, it has also been suggested to consider an evolutionary perspective to better understand differences between FTF and technology-mediated interaction (e.g., Kock, 2004; Piazza and Bering, 2009; Abraham et al., 2013). According to evolutionary psychology (e.g., Buss, 2005), human behavior can be understood from the view of evolutionary adaptations that occurred over hundreds of thousands of years in response to only slowly changing environmental conditions. With regard to technology-mediated interaction, the evolutionary perspective assumes that humans have acquired competencies in FTF communication over very many centuries while the acquisition of competencies in technology-mediated communication is only very recent in the development of humankind. Furthermore, positive social interactions with others that have occurred FTF throughout the vast majority of human evolution also serve to satisfy the underlying need for belongingness (Baumeister and Leary, 1995). However, with the advent of technology-mediated communication, interactions do not always have to be FTF, which can impair the satisfaction of social belongingness needs of the communication partners who have been used to FTF interaction throughout evolution. In line with this, Sacco and Ismail (2014), for example, found that FTF interactions satisfied the need for social belongingness of their participants better than virtual interactions or compared to a control group without interaction. With regard to selection interviews, it is therefore also conceivable that the interview medium could negatively affect the social character of these interviews because of its impact on the lower satisfaction of belongingness needs. Thus, for technology-mediated interviews, evolutionary approaches converge with predictions from the other theoretical approaches.

### Previous Research on Technology-Mediated Interviews

Most of the earlier research on technology-mediated interviews has focused on the effects of the different interview media on ratings of interviewees’ performance and on interviewees’ perceptions of the different interviews. Furthermore, most of this research focused on telephone and videoconference interviews in which an interviewer and an interviewee interacted directly. These two kinds of technology-mediated interviews are also the main focus of our study.

Concerning telephone and videoconference interviews, an obvious issue is that videoconferencing has seen considerable technological progress over the past decades, whereas fewer changes were observed for telephone interviews. This implies that research on telephone interviews that was conducted some time ago (e.g., Silvester et al., 2000; Straus et al., 2001) may still be relevant to this day. However, despite the absence of huge technological change affecting communication bandwidth, the prevalence of telephone interviews has also increased in recent years (e.g., Amoneit et al., 2020). This may be of importance as experience with telephone interviews has been identified as a moderating variable that may affect performance in these interviews (Silvester et al., 2000).

Increased familiarity with videoconferencing systems might not only affect the relevance of previous research (e.g., many students and employees are now familiar with videoconference systems like Skype, Google Hangouts, or Zoom) but also the considerable technological progress over recent years. Thus, some of the previous findings may be less relevant today because they were obtained with technology that is now considered to be obsolete (Ployhart et al., 2017). Due to rapid progress in computing power and the availability of high-speed internet and high-definition cameras, today’s communication bandwidth is considerably more extensive than it used to be 10–15 years ago. Therefore, performance impairments in videoconference interviews reported in studies published more than a decade ago should be considerably reduced when using contemporary technology. Accordingly, potential differences between FTF and videoconference interviews that were attributed to lower communication bandwidth may have become smaller, now. Nevertheless, in a series of three qualitative studies McColl and Michelotti (2019) still found that interviewers reported several limitations of videoconference interviews in comparison to FTF interviews. In addition to some remaining technical issues, these limitations included aspects such as impairments of non-verbal communication (e.g., eye contact, perceptions of hand gestures) and problems of the setting (e.g., lighting and noise).

#### Interview Performance Ratings in Technology-Mediated vs. FTF Interviews

Concerning the effects of the interview medium on interview performance ratings, a recent meta-analysis of previous studies, which have all been published at least 9 years before the meta-analysis, found that interviewees generally receive better ratings in FTF interviews than in technology-mediated interviews (Blacksmith et al., 2016, but see Chapman and Rowe, 2001, or Straus et al., 2001, for exceptions). Furthermore, the meta-analytic effects were of intermediate size and were comparable for telephone and for videoconference interviews. In addition, interviewees in previous studies also had higher outcome expectations in FTF interviews than in telephone or videoconference interviews, which means that they also considered their performance to be better in FTF interviews (Chapman et al., 2003) and assumed to be able to use more impression management in FTF interviews than in technology-mediated interviews (Basch et al., 2020a). However, despite using modern videoconference technology, two recent studies still found higher interview performance ratings (Sears et al., 2013; Basch et al., 2020b) in FTF interviews compared to videoconference interviews with mean differences of intermediate size.

A limitation of the meta-analysis by Blacksmith et al. (2016) beyond its reliance on relatively old primary studies is that the empirical basis for effects concerning specific types of interviews is also rather sparse (e.g., the meta-analytic estimate for the comparison for videoconference vs. FTF interviews is based on an *N* of only 103 individuals). In addition, the effects from the corresponding primary studies vary considerably so that specific aspects of the few primary studies and the respective interviews have more impact on the meta-analytic estimate than is usually the case in other meta-analyses. Furthermore, earlier primary studies usually also only compared FTF interviews with one kind of technology-mediated interviews (e.g., Silvester et al., 2000; Sears et al., 2013; Basch et al., 2020b). Thus, it is unclear whether the differences between FTF and telephone or videoconference interviews are indeed comparable when the content and other aspects of the interviews beyond the interview medium are held consistent. This is a relevant question, because in comparison to FTF interviews one would expect to find larger performance differences for telephone interviews than for videoconference interviews according to the different theoretical approaches because telephone interviews should go hand in hand with lower media richness, lower social presence and so on.

Another limitation of previous research is that it remains unclear whether the lower performance ratings were in fact related to lower interviewee performance or whether they were due to effects on the side of the interviewers. This issue is important in light of a study by Van Iddekinge et al. (2006) who found higher performance ratings when interviewees were rated on the basis of FTF interviews than when the same interviewees were rated on the basis of videotaped interviews. Thus, it is possible that the interview medium does not lead to lower performance by interviewees but to lower evaluations of this performance by raters.

#### Interviewee Perceptions of Technology-Mediated Interviews

Similar to the evidence regarding interview performance ratings, the majority of the previous studies also found that interviewees had a preference for FTF interviews in comparison to telephone or videoconference interviews and perceived them as fairer (e.g., Kroeck and Magnusen, 1997; Chapman et al., 2003; Sears et al., 2013). Furthermore, interviewees in Straus et al.‘s (2001) study felt more comfortable in FTF interviews than in videoconference interviews. In line with this, Blacksmith et al.’s (2016) meta-analysis found that, overall, interviewees react negatively to technology-mediated interviews in comparison to FTF interviews. Furthermore, evidence from a recent study by Basch et al. (2020b) that compared perceptions of FTF and videoconference interviews confirmed that social presence is a mediator of the effects of the interview medium on interviewees’ fairness perceptions.

The negative perceptions of technology-mediated interviews by interviewees are especially relevant for organizations because of evidence that lower fairness perceptions are accompanied by lower perceptions of organizational attractiveness (Bauer et al., 2004; Langer et al., 2020) and lower intentions to accept a job offer (Chapman et al., 2003). Thus, using technology-mediated interviews may have negative effects for the recruitment function of employment interviews (e.g., Wilhelmy et al., 2016).

Unfortunately, there are several limitations concerning previous research on interviewee perceptions of the different kinds of interviews. First, in contrast to previous research concerning interview performance, the available database related to interviewee perceptions of technology-mediated interviews is considerably smaller so that the meta-analytic results by Blacksmith et al. (2016) for the different interview media are based on an even more limited empirical basis with only two or three primary studies for each comparison. Furthermore, the primary studies that considered telephone as well as videoconference interviews either could not ensure that the interviews *per se* were comparable concerning the different interview media (Chapman et al., 2003) or the available videoconference technology that was used in the primary studies was much more plagued by impaired conversation flow in comparison to present technology (Straus et al., 2001). Finally, in some more recent studies, participants did not take part in actual interviews but only had to answer survey questions after a description of the different interviews (e.g., Basch et al., 2020a), they only observed videos of technology-mediated interviews (e.g., Langer et al., 2020), or the study did not consider telephone interviews (Basch et al., 2020b).

### Strain and Anxiety as Reactions to the Interview

Even though fairness perceptions are the most commonly investigated aspect of applicant reactions, other reactions to interviews are also relevant. For example, Straus et al.’s (2001) finding that interviewees felt more comfortable in FTF interviews than in videoconference interviews already indicated that emotional reactions might play a role when different interview media are compared.

In this context, it is important to realize that selection interviews can be a strong psychosocial stressor. This stressor may lead to interviewee strain that manifests itself not only at the subjective level (e.g., by increased anxiety) but also at the psychophysiological level (e.g., by increased transpiration and heartbeat). In line with this, stress researchers even used the analogy of an employment interview when they designed stressful situations for their research (Kirschbaum et al., 1993). Furthermore, people with higher interview anxiety show lower interview performance (e.g., McCarthy and Goffin, 2004; Powell et al., 2018), which is consistent with general evidence that applicants who experience higher test anxiety achieve lower test scores (Hausknecht et al., 2004). The latter is of particular concern in the present context because there are considerable differences between interviewees regarding interview anxiety. Thus, given that applicants are affected to different degrees by interview anxiety, this may influence the selection decision in the form of reduced chances to receive a job offer for those applicants who suffer from strong interview anxiety. Beyond the obvious negative consequences for applicants, there may also be negative effects for organizations because they may reject suitable applicants when these are unable to show their true performance level during the interview due to excessive anxiety levels (cf. McCarthy and Goffin, 2004).

There is little work on test anxiety that has examined strain beyond self-report measures. While it has not been uncommon to use psychophysiological indicators of strain in many research areas within work and organizational psychology (e.g., Åkerstedt, 1990; Zeier et al., 1996), such measures have rarely been employed in personnel selection research and none of the previous studies on technology-mediated interviews has used such indicators. Their use would allow us to obtain a broader picture of the multiple effects of stressors because it would not be necessary to rely on self-report measures and performance indicators alone. Of the many psychophysiological indicators available, heart rate variability (HRV) may be particularly suitable for the present study, as it is sensitive to changes in mental strain and negative affect (Kettunen and Keltikangas-Järvinen, 2001).

## The Present Study

The literature reviewed above has raised the central question to what extent technology-mediated interviews can influence interviewees’ performance and their perceptions of and reactions to the interview procedure. Until now, the research related to this question has mainly found that interviewees received lower performance ratings in technology-mediated interviews than in FTF interviews and that applicant perceptions of technology-mediated interviews were less positive (Blacksmith et al., 2016). However, most of the previous work was conducted at a time when a different generation of technology-mediated communication tools was used when the fidelity of the technology was much lower. At that time, interviewees were also less familiar with these communication tools. Therefore, it is unclear to what degree previous evidence can still be generalized to current applicants. Furthermore, there is a lack of research concerning strain and anxiety in the different types of interviews.

To address these issues, we set up an experiment to compare three different ways in which interviews can be conducted: In the traditional FTF manner, via telephone, or by using a videoconference system. In the latter two conditions, which used technology-mediated interviews, the interviewer and the interviewee did not meet in person. We compared the three conditions with regard to ratings of interviewees’ performance and to their perceptions of the interviews, but also with regard to the anxiety and the subjective as well as the physiological strain that they experienced.

## Methods

### Participants

A total of 95 German-speaking final year students and recent graduates from a Swiss university took part in the study. The data of 7 participants had to be discarded because they did not consent to using videotapes of their interview performance (see below). Thus, the final sample consisted of 88 participants (36 males and 52 females; age: *M* = 25.09 years; *SD* = 4.05). *Post hoc* power analyses using G^∗^Power 3.1 (Faul et al., 2009) on the basis of our sample size revealed that we had a power of 0.53 to detect a medium-sized effect (using an alpha-level of 0.05) in a one-way analysis of variance and of 0.59 for *t*-tests for mean differences between two of the three groups.

The participants were from a broad range of subjects, with larger groups majoring in communication sciences (23.9%), psychology (15.9%), and economics (12.5%). On average, participants had been enrolled for 3.82 years (*SD* = 2.09). Participants were recruited via an email that was sent to all final year students and that invited them to take part in a simulated selection interview, allowing them to gain experience regarding selection procedures and to receive feedback on their performance. All the data were collected before the advent of the COVID-19 pandemic.

### Procedure

To register for the study, participants had to complete an online registration form. After registration, they were asked to complete a questionnaire that contained questions concerning demographic and personal data. This was followed by a questionnaire measuring participants’ fairness expectations and favorability ratings for the three interview media that were examined (FTF, telephone, and videoconference). Finally, participants could make an appointment with the experimenter to come to the laboratory for the interview.

The interviews in the three experimental conditions were always conducted in the same room. This room was equipped for telephone as well as for videoconference interviews. When participants arrived in the room, they were told that the present study was investigating subjective and physiological reactions to selection procedures. Then, the heart rate monitoring system was attached to the participants’ chest and wrist. Participants were asked to sit quietly for 5 min while watching a relaxing video. The reason for this was that previous studies indicated that body movements may influence HRV measurement by introducing artificial variability (Jorna, 1992; Bernardi et al., 2000). The physiological data were collected during the last 2 min of this 5-min period and were used as a baseline for the experimental HRV measure. After the resting stage, participants completed the KAB, a short questionnaire to measure their current subjective strain levels.

Next, participants completed a short general mental ability (GMA) test. During this test, HRV data were again collected during the first 2 min of the test administration. Directly after the GMA test, participants completed the strain questionnaire for a second time.

Participants were then randomly assigned to one of the three interview conditions: FTF (*n* = 30), telephone (*n* = 26), and videoconference (*n* = 32). In all three conditions, the experimenter instructed participants to remain seated at all times (which also meant they should not get up in the FTF condition when the interviewer entered the room) to prevent contamination of HRV by movement artifacts. Furthermore, the participants were asked to imagine that they had applied for an attractive leadership position in their field of study and that they would have direct reports in this position.

After the experimenter had left the room, the actual interview began. In the FTF condition, the interviewer entered the room. To make the interview situation more authentic, the interviewer wore a suit and a tie in all conditions. For the telephone condition, a typical office telephone was used. The interview began by the interviewer calling the participant on the telephone. In the videoconference condition, a program (Skype) was used that allowed voice calls to be made over the internet with an additional videoconference function. The interviewer began the interview by calling the participant via computer, upon which a window opened on the participant’s screen, with the interviewer being shown in full screen mode on a stand-alone 22-inch computer screen. The interviewer and the interviewee used the built-in microphones to talk to each other.

To ensure that participants could have eye contact with the interviewer in the videoconference condition, the webcam that was used to record the interviewer was fixed in the middle of his screen. In contrast to this, the webcam used to record the participants was fixed on top of the screen.

At the beginning of the interview, the interviewer introduced himself. Then, the participant was asked to prepare a short self-introduction in which he/she should introduce himself/herself to the interviewer. Participants were told that this self-introduction should not last for more than 3 min and they were given 4 min to prepare their answer, which allowed us to measure HRV during the last 2 min of the preparation phase. After the self-introduction, the interviewer asked a set of standardized questions. This set consisted of seven past-behavior questions and four future-oriented questions (see below). All questions were asked in the same order and no probing or follow-up questions were used. The interviewer took notes and evaluated the participants’ answers before asking the next question.

After the interview, participants were asked for a self-evaluation of their overall interview performance. Then, they were asked to complete a questionnaire to rate their current level of interview anxiety and the perceived fairness of the interview they had just experienced, and to complete the strain questionnaire again.

During the interview, all participants were videotaped with a hidden video camera so that their performance could be evaluated by a second rater after the completion of the study. This hidden camera was necessary because in the telephone condition in particular the feeling of being observed might have changed interviewees’ perception of the situation. After the last strain questionnaire, participants were debriefed and informed that a video recording had been made. They were asked to grant permission that these video recordings can be used for later analyses. As noted above, seven participants did not grant permission so that their video recordings were deleted by the experimenter in the presence of the participant. Thus, no data from these participants were used for later analyses.

### Measures

#### Interview Performance Ratings

As noted above, the interview consisted of two parts, a short self-introduction and a set of standardized questions containing seven past-behavior questions and four future-oriented questions. The past-behavior questions asked interviewees to recall situations they had previously experienced and to describe what exactly they had done in those situations. The future-oriented questions asked interviewees to describe what they would do in hypothetical situations presented to them. Two of the questions targeted Cooperation, three targeted Information Management, five targeted Leadership, and one targeted Systematic Planning. All interview questions were taken from previous studies in which participants stem from similar populations (Melchers et al., 2009, 2012). The questions were suitable for university graduates applying for management trainee positions. An example question can be found in the Appendix.

Several ratings of interviewees’ performance were collected. First, the interviewer and a second rater (two Master students) rated interviewees’ performance in the two main parts of the interview (i.e., the self-introduction and the 11 structured interview questions). For both parts of the interview, these ratings were made on descriptively-anchored 5-point rating scales, ranging from 1 = *poor* through 3 = *average* to 5 = *good*. To ensure that both raters had a common frame of reference for their evaluations, each rating scale had descriptive anchors. These anchors were similar to BARS (Smith and Kendall, 1963) and described what behavior of an interviewee would be considered as poor, average, or good (see the example in the Appendix). The rating scales were employed in previous studies (Melchers et al., 2009, 2012) after they had undergone extensive pretesting. After the interview, the interviewer and the second rater evaluated the participants’ overall interview performance on another rating scale, again ranging from 1 = *poor* to 5 = *good.* Finally, participants also provided self-ratings of their overall interview performance on a 6-point rating scale ranging from 1 = *very poor* to 6 = *very good*.

Prior to the first interview, both raters were trained over a period of 2 days. During this training, they were introduced to the self-introduction and the different interview questions as well as to definitions and behavioral examples for answers to the different interview questions. In order to develop a consistent frame of reference for rating the interviewees’ answers, raters received specific training on the purpose of each interview question, typical errors committed by raters, and the idea behind descriptively-anchored rating scales (Melchers et al., 2011; Roch et al., 2012). Furthermore, it was emphasized that it was crucial to conduct all interviews in the same prescribed manner, that is, to read the interview questions as printed on the interview forms and not to rephrase them or give additional cues. After completing the training, one of the students was assigned the role of the interviewer during the experiment while the other one served as the second rater and also as the experimenter, who welcomed participants and looked after them.

For our later analyses, we averaged the ratings from both raters for the interviewees’ overall interview performance and for the self-introduction, respectively, and also the means from both raters across all the structured interview questions. To determine the inter-rater reliability of the ratings from the interviewer and the second rater, we calculated intraclass correlations (ICC 2,1). Mean inter-rater reliabilities (i.e., the reliability of each rater) were 0.81 (overall interview performance), 0.71 (self-introduction), and 0.96 (overall mean across the structured questions).

#### Interviewee Perceptions

We used two interviewee perception measures. First, participants rated the favorability for each of three interview media on one item (“If you applied for a job, how much would you like to go through each of the following selection procedures?”) on a scale ranging from 1 = *not at all* to 6 = *a lot*. And second, participants were asked twice to provide ratings of interview fairness. Prior to the interview, they rated the expected fairness of each interview medium (FTF, telephone, and videoconference) on a one-item 6-point rating scale (“Please rate the fairness of the following selection procedures?” ranging from 1 = *very unfair* to 6 = *very fair*). After the interview, participants rated the fairness of the interview again, but this time only the interview medium they had experienced during the study (“How fair did you feel that the experienced interview was as a selection procedure?”), using the same 6-point rating scale.

#### Strain and Anxiety

We used two ways to measure participants’ strain. First, we used the KAB (Müller and Basler, 1993), a short German-language questionnaire, to measure participants’ current subjective strain. In this questionnaire, participants had to indicate how they were feeling “right now” on six bipolar adjective pairs (e.g., tense – relaxed) using a 6-point scale. In our study, internal consistencies ranged between 0.80 and 0.86 for the different measurements.

Second, we measured heart rate variability as a psychophysiological indicator of participants’ strain. HRV describes the variation of the interval between two successive heart beats. By using spectral analysis, the main components of HRV can be separated and analyzed individually. The high frequency (HF) band represents the sympathetic activation and the low frequency (LF) band describes the parasympathetic activation (Task Force of the European Society of Cardiology and the North American Society of Pacing and Electrophysiology, 1996). The relation of these two components (i.e., the LF/HF ratio) is an indicator of psychosocial strain with a low ratio indicating elevated levels of strain (Bosch et al., 2003).

The Polar S810iTM heart rate monitor (Polar S810iTM, Kempele, Finland) was used to measure HRV in a non-intrusive manner. Specifically, each participant was equipped with a belt, containing a pulse monitor, worn around the chest. The heart rate data was transferred to a watch (worn in addition to the belt) via an infra-red connection. The data were subsequently analyzed by using the Kubios software (Niskanen et al., 2004).

In addition to strain, we also measured participants’ state anxiety during the interview. To do so, we used the MASI (Measure of Anxiety in Selection Interviews, McCarthy and Goffin, 2004) and converted the items into a state measure, which allowed us to measure the impact of interview medium on current levels of interview anxiety. The conversion involved the translation into German, but also the removal of 4 of the original 30 items because they did not apply to our study (e.g., “When meeting a job interviewer, I worry that my handshake will not be correct” because in two of the three conditions the interviewee did not meet the interviewer in person). Furthermore, changes in wording were made so that all items referred to the specific interview situation rather than the experience of being interviewed in general.

The MASI items covered aspects such as communication anxiety (e.g., “During this interview, I often couldn’t think of a thing to say”), appearance anxiety (e.g., “I often felt uneasy about my appearance during this interview”), social anxiety (e.g., “I was worrying about whether the interviewer was liking me as a person”), performance anxiety (e.g., “During this interview, I got very nervous about whether my performance was good enough”), and behavioral anxiety (e.g., “I felt sick to my stomach during the interview”). Participants rated all items on a 5-point Likert scale ranging from 1 = *strongly disagree* to 6 = *strongly agree*). In line with McCarthy and Goffin (2004), the mean across all items was calculated as an overall measure of participants’ current interview anxiety. Coefficient alpha for the MASI was 0.88.

#### Additional Variables

We used a short but extensively validated test from a consultancy to measure GMA. This test represents a commonly used measure of cognitive ability and contained 50 items to assess verbal, arithmetic, and spatial reasoning. Participants had to complete as many items as possible within 12 min.

In addition, participants provided demographic information and answered questions concerning their previous experience with face-to-face selection interviews and also with telephone and videoconference interviews. For each of these interviews, they were asked to indicate the number of previous interviews that they had experienced in the past. Furthermore, participants also had to indicate their body size and weight to determine their body-mass index and to answer questions concerning their activity levels because these variables might influence HRV.

## Results

### Preliminary Analysis

Correlations and descriptive data for all study variables are shown in Table 1. Inspection of this table shows that participants’ performance in the interview (except for the self-introduction) was significantly related to interview state anxiety and to perceived psychological strain during the interview, all *r*s > | −0.21|, all *p*s < 0.05. However, HRV was not related to interviewees’ performance.

**TABLE 1:** Descriptive information and intercorrelations for study variables.

Variable	*M*	*SD*	1	2	3	4	5	6	7	8	9	10	11	12	13	14	15	16	17	18	19	20	21
(1) Sex	0.59	0.49																					
(2) Age	25.09	4.04	−0.23*																				
(3) Interview experience	2.56	1.81	−0.22*	0.43**																			
(4) GMA test	29.77	4.38	–0.20	0.06	0.05																		
(5) Expected fairness FTF (pre-interview)	4.92	0.89	–0.02	0.00	0.03	–0.13																	
(6) Expected fairness TEL (pre-interview)	3.59	1.17	0.06	0.07	0.04	0.14	−0.22*																
(7) Expected fairness VC (pre-interview)	3.86	0.87	–0.02	0.13	0.09	0.04	0.22*	0.24*															
(8) Favorability FTF (pre-interview)	5.08	1.02	–0.07	0.21*	0.26*	0.14	0.25*	0.09	0.18														
(9) Favorability TEL (pre-interview)	3.14	1.31	–0.07	0.18	0.14	0.06	0.01	0.35**	0.09	0.05													
(10) Favorability VC (pre-interview)	3.07	1.13	0.05	0.30**	0.04	0.07	–0.17	0.13	0.11	0.08	0.39*												
(11) Perceived fairness (post-interview)	5.02	0.97	0.04	0.00	–0.09	0.05	0.00	–0.05	0.04	0.16	–0.11	–0.02											
(12) Self-introduction	4.14	0.62	–0.11	0.18	0.30**	0.01	0.05	–0.05	0.07	0.09	0.20	0.11	0.16										
(13) Standardized questions	3.85	0.46	−0.27*	0.01	0.23*	0.21	0.10	0.10	0.02	0.22*	0.19	0.00	0.18	0.25*									
(14) Overall interview rating	3.81	0.77	−0.21*	0.04	0.22*	0.19	0.14	–0.01	0.17	0.09	0.16	–0.12	0.18	0.33**	0.72**								
(15) Self-rated interview performance	4.15	0.99	−0.30**	0.04	0.06	0.14	0.24*	0.14	0.10	0.18	0.29*	0.07	0.08	0.15	0.39**	0.39**							
(16) Interview anxiety (state)	2.14	0.52	0.30**	–0.08	–0.15	–0.18	–0.05	−0.29**	–0.19	−0.25*	−0.24*	−0.22*	–0.10	–0.01	−0.27**	−0.24*	−0.46**						
(17) Psychological strain resting period	2.47	0.74	–0.07	–0.02	–0.03	0.03	–0.12	–0.02	0.00	−0.29**	–0.12	−0.21*	–0.04	0.00	0.04	0.09	–0.18	0.22*					
(18) Psychological strain GMA	3.04	0.85	0.09	–0.03	−0.26*	–0.05	–0.03	–0.11	−0.22*	–0.13	–0.02	0.00	0.01	0.10	–0.01	–0.03	–0.13	0.46**	0.31**				
(19) Psychological strain interview	2.66	0.81	0.25*	0.08	–0.10	–0.16	–0.20	–0.22	−0.33**	–0.14	–0.18	–0.13	0.02	–0.01	−0.22*	−0.29**	−0.51**	0.61**	0.12	0.42**			
(20) HRV resting period	2.20	1.45	−0.32**	0.09	0.06	0.11	0.13	–0.03	0.07	0.10	0.25*	–0.11	0.16	0.06	0.05	0.15	0.25*	–0.20	0.04	–0.06	–0.07		
(21) HRV GMA	3.25	2.32	−0.34**	0.14	0.13	0.08	0.13	–0.01	0.06	0.05	0.03	−0.24*	0.04	0.05	0.07	0.10	0.09	–0.05	0.10	–0.02	–0.13	0.61**	
(22) HRV interview	3.87	2.66	−0.31*	0.06	0.12	0.05	0.13	–0.11	–0.13	0.07	–0.12	–0.15	0.09	0.06	0.10	0.08	0.15	–0.14	0.05	–0.05	–0.09	0.47**	0.67**

N = 88. Sex was coded 0 = male, 1 = female. GMA = general mental ability; FTF = face-to-face interview; TEL = telephone-interview; VC = videoconference interview; HRV = heart rate variability. *p < 0.05 (two-tailed), ** p < 0.01 (two-tailed).

Concerning the comparability of the experimental conditions, the preliminary analyses revealed that participants in the three experimental groups did not differ with regard to age, sex, experience with technology-mediated interviews, body size, or weight, but that they differed with regard to their previous experience of selection interviews in general, *F*(2,85) = 4.60, *p* < 0.05, η^2^ = 0.10, (*M* = 3.20, *SD* = 1.88, for the FTF condition, *M* = 4.42, *SD* = 1.94, for the telephone condition, and *M* = 3.19, *SD* = 1.38, for the videoconference condition). Therefore, we used interview experience as a covariate for all later analyses that compared the three experimental groups.

### Interview Performance Ratings

Means and *SD*s for ratings of participants’ performance in the different interview conditions as well as effects sizes for the mean differences are shown in Table 2. Descriptively the means in the FTF condition were higher than the means in the two other conditions for all the different ratings of participants’ interview performance. Furthermore, one-factorial ANCOVAs with prior interview experience as a covariate also revealed a significant main effect of the interview condition on interview performance ratings for the standardized questions, *F*(2,84) = 5.15, *p* < 0.01, η^2^ = 0.11, and for the overall interview ratings, *F*(2,84) = 3.36, *p* < 0.05, η^2^ = 0.07. Pairwise comparisons confirmed that the significant main effects were driven by higher performance ratings in the FTF condition in comparison to the other two conditions. Most of the differences were in the medium to large range (cf. Table 2). The ANOVA for interviewees’ self-rated overall performance was not significant, *F*(2,84) = 2.70, *p* > 0.05, η^2^ = 0.06, even though the mean differences were still in the medium range (cf. Table 2). Finally, given the small mean differences, the main effect for performance ratings in the self-introduction failed to reach significance, *F* < 1.

**TABLE 2:** Means, standard deviations, and Cohen’s *d*s for the different interview conditions.

	Interview medium								
	Face-to-face (*n* = 30)		Telephone (*n* = 26)		Videoconference (*n* = 32)		Cohen’s *d*		
	*M*	(*SD*)	*M*	(*SD*)	*M*	(*SD*)	FTF-Tel	FTF-VC	Tel-VC
**Interview performance:**									
Self-introduction	4.21	(0.61)	4.08	(0.63)	4.13	(0.61)	0.21	0.13	−0.08
Standardized questions	4.04	(0.43)	3.65	(0.45)	3.83	(0.43)	0.89*	0.49*	−0.41
Overall interview rating	4.09	(0.74)	3.70	(0.76)	3.63	(0.74)	0.52	0.62*	0.09
Self-rating overall performance	4.49	(0.98)	3.99	(1.01)	3.96	(0.98)	0.50	0.54*	0.03
**Interviewee perceptions:**									
Favorability (pre-interview)^*a*^	5.08	(1.02)	3.14	(1.31)	3.07	(1.13)	1.19**	1.38**	0.05
Expected fairness (pre-interview)^*a*^	4.92	(0.89)	3.59	(1.17)	3.86	(0.87)	0.82**	0.96**	−0.21
Perceived fairness (post-interview)	5.26	(0.97)	4.79	(1.00)	4.99	(0.97)	0.48	0.28	−0.20
**Strain and anxiety:**									
Subjective strain	2.49	(0.80)	2.56	(0.83)	2.89	(0.80)	−0.09	−0.50	−0.41
Heart rate variability	3.32	(2.67)	4.46	(2.75)	3.91	(2.68)	−0.42	−0.22	0.20
Interview anxiety (state)	2.08	(0.52)	2.09	(0.53)	2.22	(0.52)	−0.02	−0.27	−0.25

Entries refer to adjusted means using interview experience as a covariate for all between-group comparisons. Asterisks indicate whether a specific comparison was significant according to pairwise comparisons. ^*a*^Means and SDs for the pre-interview perceptions variables are based on all 88 participants and the corresponding effect sizes represent Cohen’s ds for dependent variables. *p < 0.05, **p < 0.01 (two-tailed).

In addition to these analyses, we also conducted separate ANCOVAs for the ratings from the interviewer and those from the second rater. This was done because of findings by Van Iddekinge et al. (2006) that ratings from a FTF interview were significantly higher than ratings of the same interviews on the basis of videotapes. The additional ANCOVAs generally paralleled the previous results, with the exception that the analysis with the overall interview rating was no longer significant for the second rater, *F*(2,84) = 2.87, *p* > 0.05, η^2^ = 0.06. All other ANCOVAs remained significant. Furthermore, we also compared ratings from the interviewer with those from the second rater for the FTF condition directly because this is the same kind of comparison as in Basch et al.’s (2020b) and Van Iddekinge et al.’s (2006) studies. None of these comparisons reached significance, all *ts* < 1.48, all *ps* > 0.05. Thus, the overall pattern of results suggested that the differences between the interview conditions were related to performance differences between interviewees in the different conditions and not to differences in rating levels or rater biases between the interviewer and the second rater.

### Interviewee Perceptions

The perceptions of the different interview media are also shown in Table 2. The means showed a clear preference for FTF interviews over both types of technology-mediated interviews. In line with this, a one-factorial repeated-measures ANOVA for participants’ interview favorability ratings revealed a significant difference, *F*(2,174) = 104.88, *p* < 0.01, η^2^ = 0.55. The same pattern was also observed for expected fairness prior to the interview experience, with higher ratings for the FTF interview over the two other interviews, *F*(2,174) = 47.63, *p* < 0.01, η^2^ = 0.35. As for interview performance, these main effects were again driven by the more positive attitudes toward FTF interviews and the corresponding mean differences represent large effects (cf. Table 2).

Interestingly, after participants had actually experienced their respective interview, the difference in fairness ratings was no longer significant, as was revealed by a one-factorial ANCOVA, *F*(2,85) = 1.88, *p* > 0.05. The corresponding effect sizes were in the low to medium range (cf. Table 2). The reason for this was that perceived fairness scores after the interview were considerably higher in comparison to pre-interview fairness expectation for the telephone and videoconference conditions, *t*(25) = 3.55 and *t*(31) = 6.47, both *p*s < 0.01, Cohen’s *d*s = 0.70 and 1.14, respectively. Thus, actually experiencing these technology-mediated interviews seems to have alleviated some of the interviewees’ previous concerns. In contrast, only a minor and non-significant difference was found for the comparison of pre- vs. post-interview fairness perceptions for the FTF condition, *t* < 1, *d* = 0.15.

### Strain and Anxiety

In a first step, we tested whether the two strain variables (i.e., the self-report measure as an indicator of interviewees’ subjective strain and the HRV as an indicator of their physiological strain) would reflect expected differences between the resting stage on the one hand and the GMA test and the interview, respectively, on the other hand. Descriptive data for the different experimental stages can be found in Table 3.

**TABLE 3:** Subjective and physiological strain during different stages of the selection simulation.

	Resting stage		GMA test		Interview	
	*M*	(*SD*)	*M*	(*SD*)	*M*	(*SD*)
Subjective strain	2.47^*a*^	(0.74)	3.04^*b*^	(0.85)	2.66^*a*^	(0.81)
Heart rate variability	2.20^*a*^	(1.45)	3.25^*b*^	(2.32)	3.87^*c*^	(2.66)

**N* = 88. Means in the same row that do not share a common superscript differ according to pairwise comparisons (*p* < 0.05, two-tailed).*

For both variables, interviewees’ subjectively experienced strain and their HRV, we found the lowest means during the resting stage (cf. Table 3). In line with this, one-factorial repeated-measures ANOVAs revealed a significant main effect of the experimental stage, *F*(2,174) = 16.08, *p* < 0.01, η^2^ = 0.16, for subjective strain and *F*(2,174) = 28.46, *p* < 0.01, η^2^ = 0.25, for HRV.

In the next step, we compared interviewees’ subjective strain between the different interview conditions. As can be seen in Table 2, there were only rather small descriptive differences in participants’ subjective strain. In line with this, the interview condition was neither significant in the one-factorial ANCOVAs for subjective strain, *F*(2,84) = 2.26, *p* > 0.05, η^2^ = 0.05, nor for interview state anxiety, *F* < 1, η^2^ = 0.02. Furthermore, even though HRV was lowest in the FTF condition, this difference also failed to reach significance, *F*(2,84) = 1.20, *p* > 0.05, η^2^ = 0.03.

## Discussion

The aim of the present study was to evaluate whether the interview medium affects ratings of interviewees’ performance in a simulated selection interview, their perceptions of the interview, and the strain and anxiety that they experience during the interview. It makes several contributions to the literature. First, it confirms results from previous studies that were conducted in the previous decade when technology-mediated interviews were less common and videoconference systems were less powerful than today (Blacksmith et al., 2016). In line with these earlier findings and with the different communication-based theories and with the evolutionary perspective, we found that interviewees obtained lower performance ratings in technology-mediated interviews and had less positive views of these interviews before the interview. Thus, the interview medium still makes a difference for current interviewees who are more used to using communication technologies.

Furthermore, the differences of performance ratings were particularly strong for answers to the standardized questions, which included past behavior as well as future-oriented questions, but were negligible for the self-introduction. This may be because asking standardized questions resulted in an ongoing interaction, which required continuous adjustments of the interviewee. No such adjustments were required during the self-introduction because it involved a monolog. Alternatively, the preparation time that was granted to interviewees to prepare for the self-introduction was so long that interviewees could prepare their answer in sufficient detail so that potential effects of the interview medium no longer impacted the quality of their answers.

As a second contribution, we did not find any significant differences between telephone vs. videoconference interviews and descriptively the differences between them and FTF interviews were sometimes larger for telephone interviews and sometimes for videoconference interviews. Based on media richness theory (Daft and Lengel, 1986), social presence theory (Short et al., 1976), and Potosky’s (2008) framework of media attributes we would have expected larger differences for telephone interviews than for videoconference interviews. This suggests that the two technology-mediated interview media were more alike concerning the effects they produced than assumed by the different theoretical approaches.

As a third contribution to the existing literature, we found large effects concerning initial differences between interviewees’ perceptions of the different interviews but also that these differences were no longer significant once interviewees had completed their respective interview. This is an important finding given that participants in some previous studies did not experience actual interviews (e.g., Basch et al., 2020a) whereas they did in others (e.g., Chapman et al., 2003). Concerning the pre-interview results, we found that interviewees expressed more positive opinions on FTF interviews than on both technology-mediated interviews prior to the interview. Qualitatively, this replicates the overall pattern of results from previous studies (e.g., Kroeck and Magnusen, 1997; Chapman et al., 2003). Furthermore, our pre-interview results suggest that stronger familiarity with new technologies among current participants does not lead to more favorable initial views of these interviews. Given that fairness perceptions of a selection procedure can influence important outcomes like applicants’ perceptions of organizational attractiveness (Hausknecht et al., 2004), reapplication behavior (Gilliland et al., 2001), or job offer acceptance (Harold et al., 2016), they can affect the recruitment function of the interview. Thus, when organizations use an interview medium for which applicants hold lower pre-interview perceptions then this might impair perceptions of organizational attractiveness so that applicants are less likely to accept an invitation for such an interview. However, after having experienced the interview, no significant differences were found any more. This suggests that actual experience can alleviate concerns among interviewees with regard to telephone or videoconference interviews. Thus, the recruitment function of the interview is mainly at risk during the pre-interview stage.

As a final contribution, we extended the usual set of dependent variables and also investigated potential differences between the different interview media concerning strain and anxiety. However, we found no significant differences between the different interview media for the respective variables. Thus, in contrast to the performance ratings and the perception variables, interviewees did not experience differences in strain levels as a function of the interview medium and this was observed for the psychophysiological data as well as for subjective strain. Similarly, the data for state anxiety did not provide any evidence for an influence of the interview medium, either. This converging evidence from three measures suggests that neither the technology itself nor the setting associated with the two technologies had a stronger negative effect on participant strain than FTF interviews did. Thus, it seems unlikely that differences in participants’ strain or anxiety led to the observed differences for the performance ratings. While there were no differences between the three interview conditions, there was clear evidence from the physiological data as well as the self-report data that interviews caused strain among participants, compared to pre-interview baseline levels. Furthermore, the general finding that state anxiety as well as subjective strain was negatively related to interview performance ratings indicates that participants differed with regard to anxiety and strain and that these differences were related to their performance.

In addition to the different contributions, the present study also avoided several limitations of earlier research. First, we evaluated effects of the interview medium while at the same time holding the content of the interview constant. Thus, differences were indeed related to differences in the interview medium. And second, as noted above, Van Iddekinge et al. (2006) found that interviewees received higher performance ratings when they were rated on the basis of FTF interviews than when they were rated on the basis of videotaped interviews. A limitation of previous studies on technology-mediated interviews is that they could not rule out that higher ratings for FTF interviews in comparison to technology-mediated interviews were not due to better performance by interviewees but due to biases on the side of the interviewer. However, the present results suggest that interviewees indeed performed worse in technology-mediated interviews.

### Limitations and Lines for Future Research

A potential limitation of the present study concerns the generalizability of our results. As the current study was a lab-based interview simulation one might question whether the present results are also representative of high-stakes selection situations. Although our data suggest that participants experienced the interviews as stressful so that HRV was higher during the interview than for the baseline condition, this increase does not mean that the situation in our study is equivalent to a high-stakes field setting. However, as interviewees volunteered to take part in the study with the aim to gain first-hand experience of job interviews, there is reason to believe that they considered the procedure as a serious dry-run for a real job interview. This would also support evidence from other studies that used a similar approach and in which the vast majority of the participants indicated that they had acted and felt as in a real selection interview (e.g., Van Iddekinge et al., 2005; Jansen et al., 2013; Oostrom et al., 2016). Nevertheless, using a student sample may underestimate the differences between interview conditions since familiarity with modern communication technologies such as videoconferencing may have contributed to a lessening of concerns related to these technologies. Therefore, differences in performance and perceived fairness may be more pronounced for older or non-academic job applicants. Furthermore, applicant reaction research has often revealed that real applicants show more pronounced responses in comparison to participants from simulated settings (e.g., Hausknecht et al., 2004; Truxillo et al., 2009). Thus, future research with real applicants is crucial to evaluate whether potential effects in high-stakes selection contexts are larger than in our study. However, it might also be possible that the need to use videoconference technologies during the COVID-19 pandemic in many jobs – and also more experience with technology-mediated interviews for people who were on the job market during the pandemic – changed perceptions of technology-mediated interviews. Therefore, it would be good to repeat the present study once the COVID-19 pandemic is over.

A second limitation concerns the size of our participant sample. As explained above, the current sample size led to only moderate levels of power. And even though many of our analyses revealed significant differences between FTF interviews on the one hand and technology-mediated interviews on the other hand, we have to acknowledge that the sample was probably too small to detect potential differences between telephone vs. videoconference interviews if the true differences are small in magnitude.

As a third limitation, we want to mention the missing convergence of interview anxiety and strain as self-report variables and HRV as a physiological indicator. Even though it is not uncommon that self-ratings of variables like anxiety or strain do not closely correspond to other indicators of the same constructs, (e.g., Feiler and Powell, 2013) future research should consider whether other physiological markers of strain (e.g., cortisol levels) match self-report measures more closely.

Fourth, we only used one-item measures for some of the interviewee perception variables. The reason for this was to alleviate participants’ necessary effort to complete our surveys. Nevertheless, it would have been beneficial to use multi-item measures to improve the reliability of the corresponding measures.

And a fifth limitation concerns the issue that the present research provides only limited insights into the reasons that contributed to the performance differences between the different interview conditions. As noted above, our results are at variance with the different communication-based theories. However, a direct test in which central variables assumed by the theories such as social presence, for example, are measured might allow better insights.

In addition to these limitations another potential line for future research might be to also consider interviewer perceptions of the different interview media. Specifically, it would be interesting to evaluate whether interviewers’ preferences for the different interview media converge with interviewees’ preferences. As already noted above, the interview medium might impair additional interview functions beyond selection and assessment and some of these functions might be more salient to interviewers. Furthermore, especially in unstructured interviews, the intended assessment of fit between the organization’s or the interviewer’s values and applicants’ personal values is also relevant for many interviewers (Adams et al., 1994) and it might well be that interviewers might consider technology-mediated interviews as less suitable for this. In contrast, the potential flexibility-related advantages of technology-mediated interviews might be more salient for interviewers and this might be especially true for interviewers who interview large numbers of applicants. However, until now information on interviewers’ views of technology-mediated interview are largely missing. Therefore, future research is needed to consider interviewers’ perceptions of the different media and also whether there are conditions that influence these perceptions such as the use or non-use of structured interviews or the number of interviews that an interviewer conducts per year.

### Practical Implications

Considering the different performance ratings between the different interview conditions we recommend that the same interview medium is used for all applicants. Thus, although it might facilitate organizational selection processes if different interview media are used in the same selection process (e.g., so that applicants in the vicinity are interviewed face-to-face and those living further away by videoconference or telephone), this might increase variance in interviewer ratings that is not related to interviewees’ actual performance because according to our results interviewee performance ratings were affected by the interview medium. Therefore, the interview medium might even be added to the list of factors (cf. Campion et al., 1997; Levashina et al., 2014) that should be standardized across applicants.

Conversely, for applicants it seems advisable that they choose the former if they are offered the possibility to choose between a FTF interview and a telephone or videoconference interview. Taking part in a FTF interview seems to be beneficial for applicants’ interview performance. Thus, it might increase their chances for a job offer.

Furthermore, organizations and recruiters should be aware of applicants’ concerns about technology-mediated interviews. Even though these concerns were alleviated in the present study after participants had experienced the actual interview, their initial preferences and fairness expectations clearly favored FTF interviews. Since evidence has shown that such expectations can affect several other important perceptions and intentions (Bell et al., 2006), organizations might have to pay a price that impairs the recruitment function of their interviews if they ignore applicants’ preferences for FTF interview. Fortunately, however, previous research on the effects of explanations (Truxillo et al., 2009) suggests that explanations that provide a justification concerning the use of a selection procedure or that stress its advantages are often a suitable way to address applicants’ concerns. In line with this research, recent evidence has shown that such explanations can also improve perceptions of technology-mediated interviews (Basch and Melchers, 2019).

As organizations will probably make increasing use of technology-mediated interviews (and probably especially of videoconference interviews) in the future given the ongoing technological progress and also given the experience of the COVID-19 pandemic, an additional general issue for applied settings concerns the question whether the interview medium affects the psychometric properties of the interview. Even though our study does not allow conclusions in this regard, there is evidence that structured technology-mediated interviews can be valid predictors of interviewees’ job performance (e.g., Schmidt and Rader, 1999; Gorman et al., 2018). Thus, it seems likely that the same factors as for FTF interviews contribute to the validity of these technology-mediated interviews. Especially the usual aspects of structure seem relevant in this regard such as standardized questions for all applicants or the use job-related questions and of common frame-of-reference to evaluate applicants’ answers.

## Data Availability Statement

The data supporting the conclusions of this article can be obtained from the first author.

## Ethics Statement

Ethical review and approval was not required for the study on human participants in accordance with the local legislation and institutional requirements. The participants provided their written informed consent to participate in this study.

## Author Contributions

AP designed the study together with KM and JS. AP conducted the study. KM analyzed the data and drafted the manuscript. JB and JS edited and revised previous versions of the manuscript. JS provided feedback during all stages of the study. All authors contributed to the article and approved the submitted version.

## Conflict of Interest

AP is employed by Migros-Genossenschafts-Bund, Zurich, Switzerland. The remaining authors declare that the research was conducted in the absence of any commercial or financial relationships that could be construed as a potential conflict of interest.

## APPENDIX

### Appendix

Example of a past-oriented interview question used in the interview and of the corresponding descriptive anchors:

“You probably remember days like this: Your day was packed with appointments, for example school, sports, doctors’ appointments, and you have invited friends over for a dinner at your place but still needed to do the grocery shopping. Furthermore, there were several important phone-calls to be made that couldn’t be delayed. How did you deal with that situation?”

Descriptive anchors for 5 = *good* through 3 = *average* to 1 = *poor* answers:

5 (good): set priorities and made a written daily plan. Thought about how tasks could be summarized in a meaningful way (e.g., doctor’s office is next to the gym).
3 (average): thought about an order in which he/she wanted to do things and kept to it to a large extent.
1 (poor): simply worked through the tasks in a more or less spontaneous manner.
